# Long noncoding RNA MEG8 induces an imbalance of Th17/Treg cells through the miR-107/STAT3 axis in Henoch-Schonlein purpura rats

**DOI:** 10.18632/aging.205266

**Published:** 2023-12-04

**Authors:** Mingyu Jiang, Jicheng Dai, Chunming Jiang, Yanbo Pan, Mingyong Ren, Mengnan Xing

**Affiliations:** 1 Department of Pediatrics, The First Affiliated Hospital of Harbin Medical University, Harbin 150001, P.R. China; 2 Department of Neonatology, Zhuhai Women and Children’s Hospital, Zhuhai 519060, P.R. China; 3 Department of Neurosurgery, Tieling Central Hospital, Tieling 112000, P.R. China

**Keywords:** Henoch-Schonlein purpura, maternally expressed gene 8, miR-107, signal transducer and activator of transcription-3, Th17/Treg imbalance

## Abstract

T-helper (Th) 17/ T-regulatory (Treg) cell dysregulation underlies the pathogenesis of Henoch-Schonlein purpura (HSP). This research focused on the implication/s of the long noncoding RNA (lncRNAs) maternally expressed gene 8 (MEG8) in Th17 and Treg cell differentiation in HSP rats. MEG8, miR-107, signal transducer and activator of transcription-3 (STAT3), receptor-related orphan receptor γt (RORγt), and the transcription factor forkhead box P3 (Foxp3) expression levels were detected using real-time quantitative polymerase chain reaction and Western blot analyses. Flow cytometry was employed for measuring Th17 and Treg cells within the CD4+ T cell population. The interaction between miR-107 and MEG8 or STAT3 was examined. A low proportion of MEG8 and Treg cells together with Th17 cells were denoted within HSP rats. Moreover, MEG8 overexpression altered the Th17/Treg imbalance in peripheral blood CD4+ T-cell population, and the miR-107 mimic and STAT3 silencing reversed this effect. Thus, MEG8 served as a sponge for miR-107, lowering binding activity to STAT3 and thus overexpressing the molecule. Taken together, MEG8 induces an imbalance of Th17/Treg cells through the miR-107/STAT3 axis in HSP rats.

## INTRODUCTION

Henoch–Schonlein purpura (HSP) is a systemic vasculitis that typically occurs during childhood. To date, the underlying pathogenesis has not yet been clarified. It might involve humoral immune disorder, T-cell subset dysfunction, abnormal cytokine secretion, coagulation disorder, and fibrinolysis [1]. Interleukin (IL)-17–secreting T helper (Th17) cells, together with CD4+/CD25+ regulatory T (Treg) cells, are derived from Naive CD4+ T cells. Such T-lymphocyte subsets possess proinflammatory or anti-inflammatory functions that are interrelated regarding differentiation though are antagonistic in function and are crucial for maintaining the body’s immune balance [2]. The Th17 cell population represents a CD4+ T-cell-derived subset producing IL-17. The specific transcription factor is receptor-related orphan receptor γt (RORγt), which affects the inflammatory or autoimmune response. Treg cells consist of a T-cell subset with an immunosuppressive function that effectuates transplantation rejection and regulates autoimmune diseases. Transcription factor Foxp3 in Treg cells is expressed at higher levels. It is a characteristic marker and a specific transcription factor of Treg cells [3]. Chen et al. [4] demonstrated Th17/Treg cells ratio dysregulation is intimately linked with various autoimmune and infectious conditions as well as cancer. It is also speculated that overactivation of Th17 cells and a decline in the proportion of Treg cells causes an immunosuppressive effect, eventually leading to immune dysfunction in HSP [5]. This provides a novel therapy for HSP.

Long noncoding RNAs (lncRNAs) exhibit various physiological functions and play a regulatory role in many vital areas such as epigenetics, cell cycle, and differentiation [6]. lncRNAs can affect the development and differentiation of immune cells and maintain the homeostasis of the immune system [7]. The CD4+ T-cell population participates in host immune responses with respect to organismic development, differentiation, and activation, which are regulated by lncRNAs [8–10]. Recently, Lei et al. [11, 12] reported an association of the MEG8/miR-378d/SOBP (lncRNA–miRNA–mRNA) network with the progression or prognostic outcomes of ovarian cancer through Th17/Treg immune responses in the cytokine pathway. However, whether MEG8 participates in the regulation of Th17/Treg in HSP remains to be elucidated. Thus, MEG8 mechanistics for regulating Th17/Treg dysregulation within HSP was studied, that would provide potential new targets for HSP treatment.

## RESULTS

### Low expression of MEG8 and Treg cells in HSP rats

**Figure 1:**
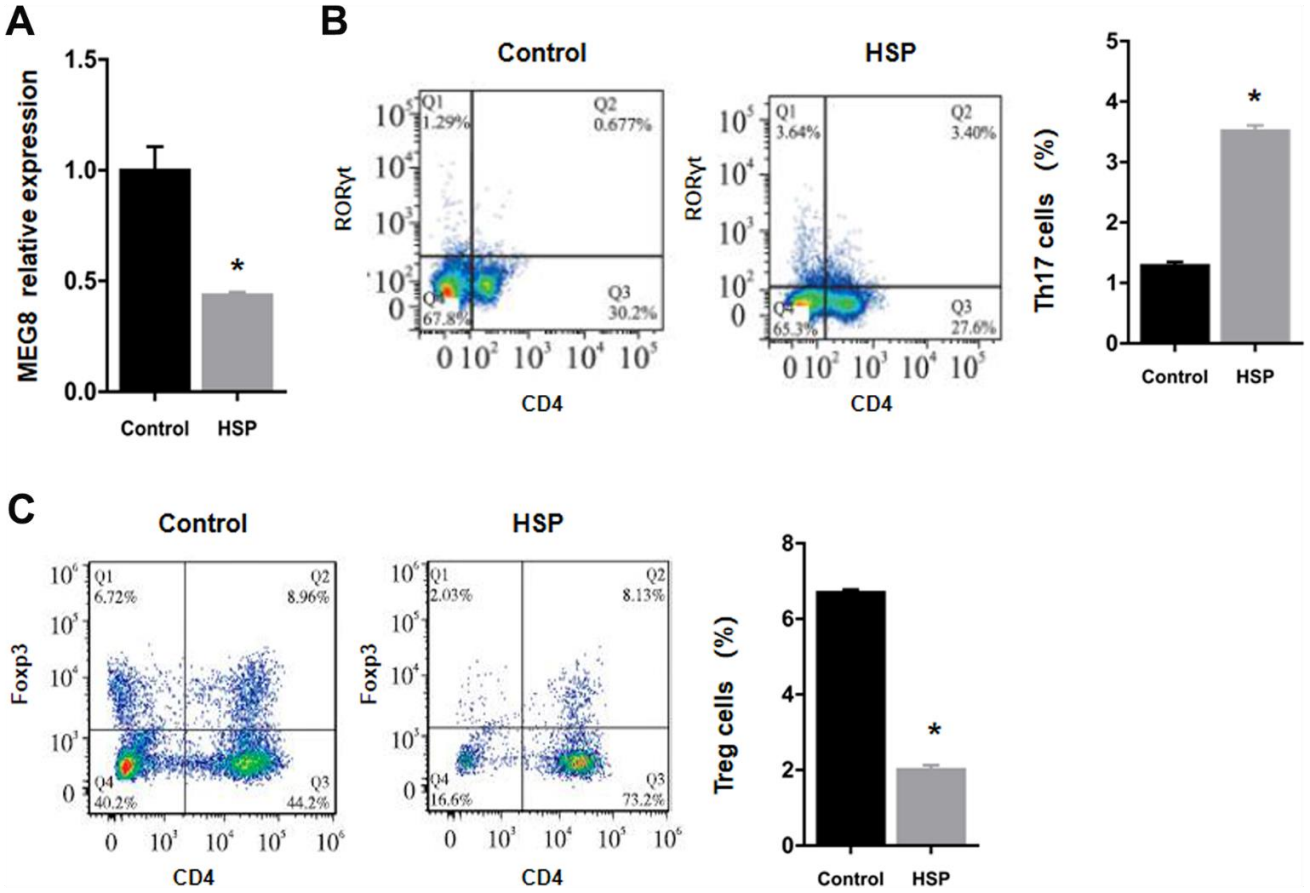
**Downregulation of the expression of MEG8 and Treg cell presence within HSP rats.** (**A**) Determination of the expression of MEG8 in peripheral blood by qRT-PCR. (**B**, **C**) The percentages of Th17 and Treg cells were determined in PBMCs derived from control rats (n = 20) and HSP rats (n = 20). ^*^P < 0.01 vs. Control group.

MEG8 demonstrated severe downregulation within the HSP group rat peripheral blood in comparison to the control group (Figure 1A). Flow cytometry findings revealed that the subpopulation number was increased for Th17 cells, but decreased for Treg cells in HSP-derived rat monocyte–derived macrophages (RMDMs) (Figure 1B, 1C).

### Overexpression of MEG8 promotes Treg differentiation in CD4+ T cells of HSP rats

**Figure 2:**
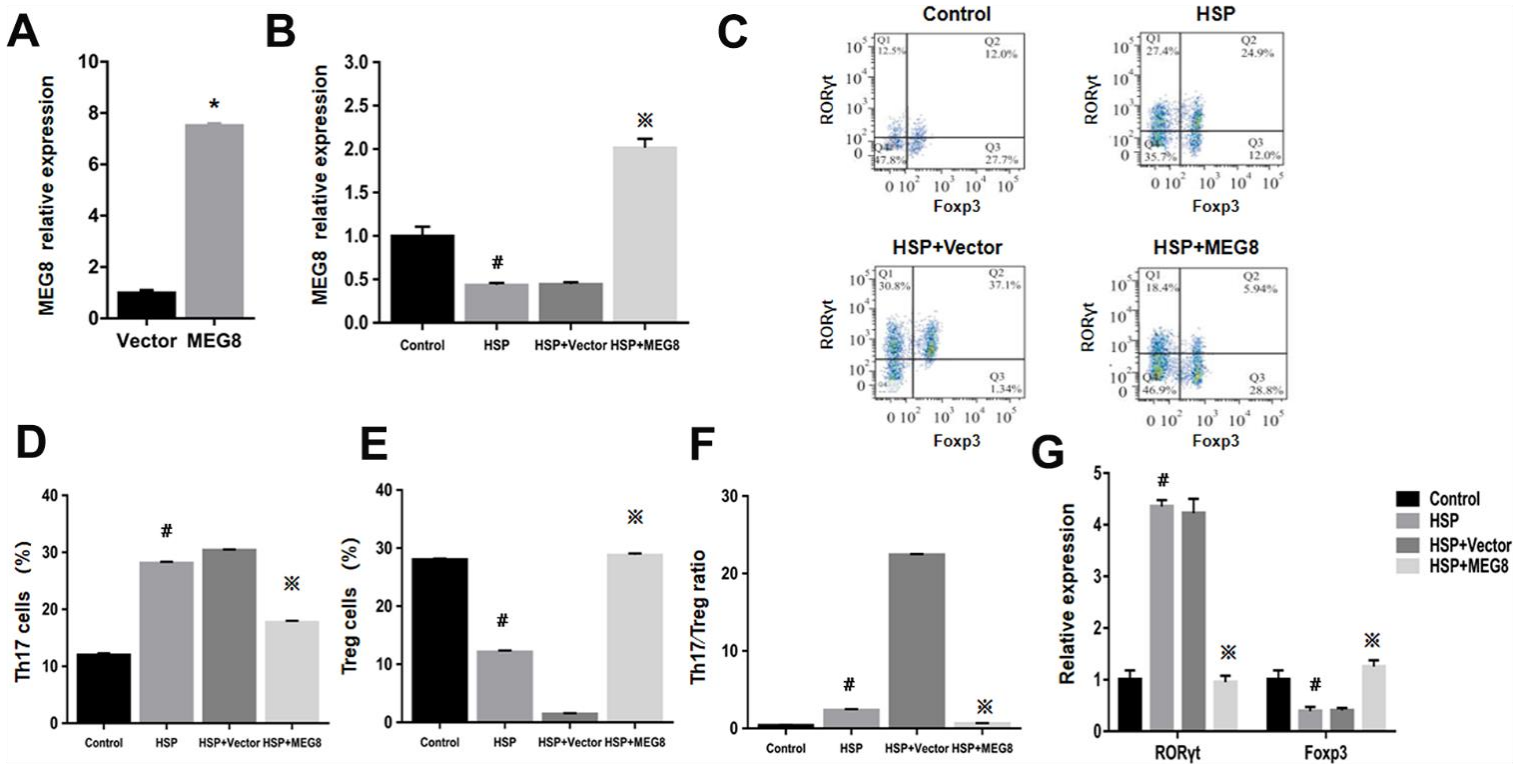
**The overexpression level of MEG8 promotes the differentiation of Treg cells in CD4+ T cells of HSP rats.** (**A**) RT-qPCR analysis of MEG8 in peripheral blood CD4+ T cellular populations derived from control groups transfected with the empty vector (Vector group) or MEG8 over-expression vector (MEG8 cohort). (**B**) MEG8 was analyzed through RT-qPCR. (**C**–**F**) Th17/Treg cellular population analysis. (**G**) The analysis of peripheral blood CD4 + T cells, HSP + carrier group or HSP + MEG8 group in the control and HSP rats through qRT-PCR. ^*^P <0.01 vs. Vector group; ^#^*P <*0.01 vs. Control group; ^※^*P <*0.01 vs. HSP+ vector group.

MEG8 function within Th17/Treg ratio regulation for HSP was examined by constructing an MEG8 overexpression vector, which was transfected into peripheral blood CD4+ T cells derived from the control and HSP groups (Figure 2). The efficiency of MEG8 overexpression in CD4+ T cells from HSP rats was significantly reduced compared to the controls (Figure 2A). MEG8 was upregulated obviously in CD4+ T cells in HSP rats (Figure 2B). However, the overexpression of MEG8 reversed the Th17/Treg ratio imbalance in CD4+ T cells of HSP rats. Furthermore, a higher proportion of Th17 cells (Figure 2C, 2D), a lower proportion of Treg cells (Figure 2C, 2E), and a higher proportion of Th17/Treg cells (Figure 2F) were observed compared to the controls. Also, the mRNA level of *ROR-γt* was upregulated, while that of *Foxp3* was downregulated in CD4+ T cells of HSP rats. (Figure 2G).

### MEG8 knockdown suppressed Treg differentiation in CD4+ T cells from HSP rats

**Figure 3:**
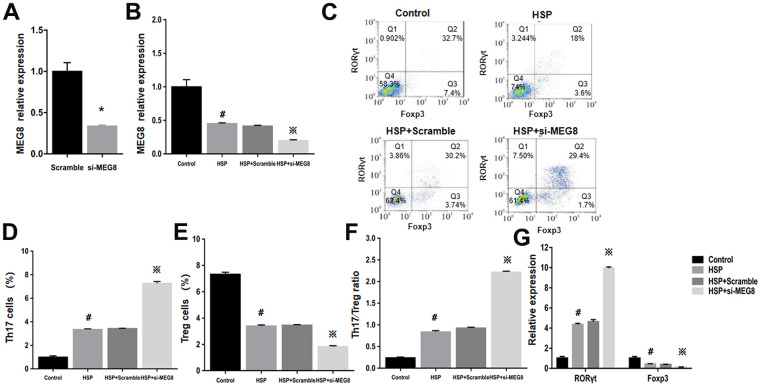
**Deletion of MEG8 inhibited the differentiation of the Treg population in HSP CD4+ T cells.** (**A**, **B**) MEG8 expression in the control group transfected with scramble and si-MEG8 was analyzed through qRT-PCR. (**C**–**F**) Analysis of Th17/Treg cells, and (**G**) RT-qPCR examination for RORγt/Foxp3 expression (control group), peripheral blood CD4+ T cells (HSP group), (HSP + scramble) or si-MEG8 (HSP + si- MEG8). ^*^P <0.01 vs. Scramble group; ^#^*P <*0.01 vs. Control group; ^※^*P <*0.01 vs. HSP + Scramble group.

Knockout effectiveness was confirmed within the control group (Figure 3A). Compared with control cells, significant MEG8 downregulation occurred within CD4+ T cells from HSP rats. Knockdown further downregulated MEG8 within HSP CD4+ T cell group (Figure 3B). The degree (%) for Th17 and Treg cellular populations within the HSP CD4+ T-cell population (Figure 3C–3E), Th17/Treg population ratio (Figure 3F), together with RORγt and Foxp3 mRNA levels (Figure 3G) were similar to those shown in Figure 2. However, MEG8 deletion exacerbated the Th17/Treg ratio disequilibrium within HSP CD4+ T cells.

### MEG8 sponges miR-107 to cause STAT3 overexpression

**Figure 4:**
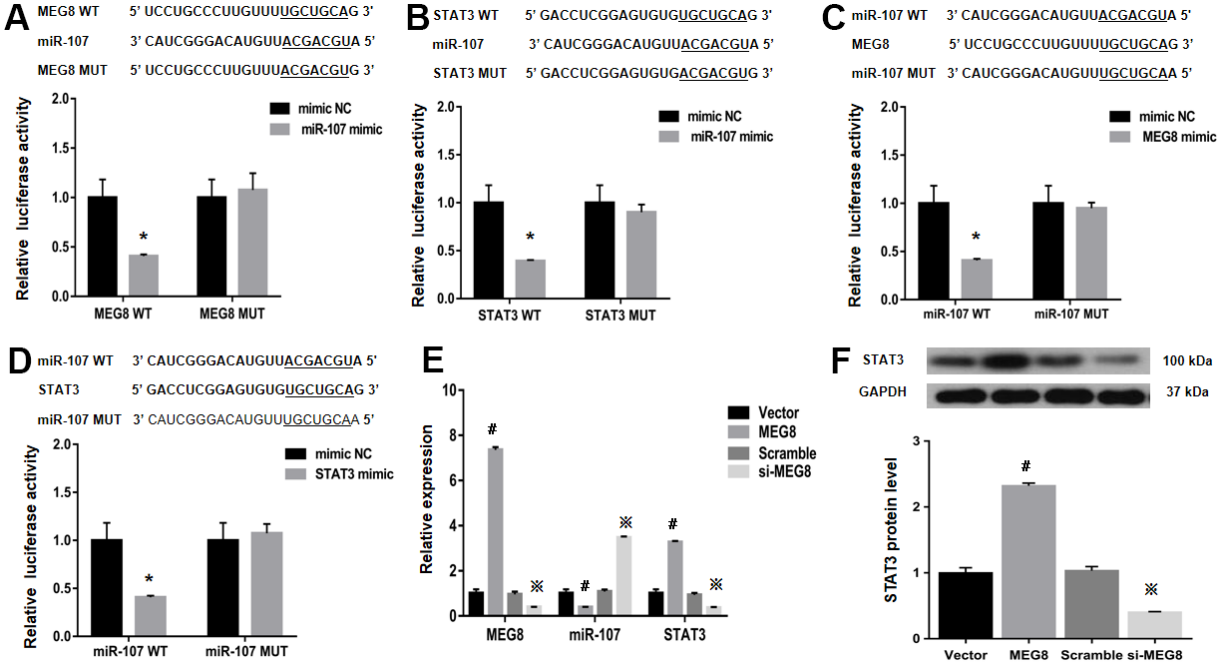
**Examination of luciferase activity in NR8383 cells among MEG8, miR-107, and STAT3 expression.** (**A**) MEG8 WT and MEG8 Mut in the bonding locations for MEG8/miR-107. (**B**) STAT3 WT and STAT3 Mut of bonding locations for STAT3/miR-107. (**C**) miR-107 WT and miR-107 Mut in bonding locations for MEG8/miR-107, (**D**) STAT3 and miR-107. (**E**) MEG8, miR-107, and STAT3 expression. (**F**) Analysis of the STAT3 protein levels within control CD4+ T cells transfected with the MEG8 overexpression vector, empty vector, si-MEG8 or scramble siRNA. ^*^P <0.01 vs. mimic NC group; ^#^*P <*0.01 vs. Vector group; ^※^*P <*0.01 vs. Scramble group.

**Figure 5:**
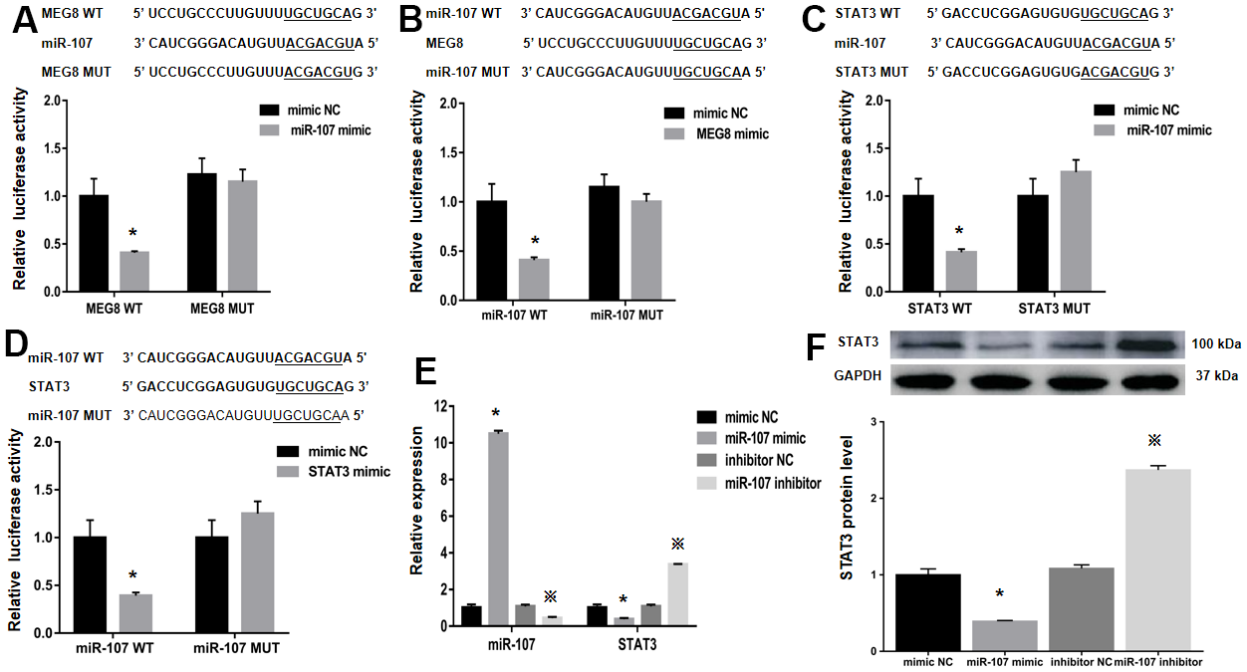
**The relative luciferase function of miR-107, MEG8 and STAT3 expression in RAOEC cells.** (**A**) The MEG8 WT and MEG8 Mut in bonding locations for MEG8/miR-107. (**B**) The miR-107 WT and miR-107 Mut of bonding locations for MEG8/miR-107. (**C**) The STAT3 WT and STAT3 Mut. (**D**) miR-107 WT and miR-107 Mut. (**E**) miR-107 and STAT3 expression, and (**F**) STAT3 proteomic content in control CD4+ T cells transfected with the miR-107 mimic, mimic NC, miR-107 inhibitor, and inhibitor NC. *P <0.01 vs. mimic NC group; ^※^*P <*0.01 vs. inhibitor NC group.

StarBase was employed to identify binding sites between MEG8 and miR-107 to assess the molecular mechanisms underlying MEG8-mediated Treg differentiation (Figure 4A). It is identified that STAT3, a transcription factor that can promote Treg differentiation, harbors an miR-107-bonding sequence within its 3′-untranslated region (UTR; Figure 4B). The miR-107 mimic effectively lowered luciferase function within the MEG8 WT group in NR8383 cells (Figure 4A). Also, MEG8 reduced luciferase function within the miR-107 WT cohort (Figure 4C), thereby indicating miR-107 / MEG8 interplays. The MiR-107 mimic also successfully lowered luciferase function of STAT3 WT reporter (Figure 4B) and miR-107 WT reporter after stimulation with STAT3 (Figure 4D) in NR8383 cells, indicating that miR-107 directly targeted the 3′-UTR of STAT3. Furthermore, MEG8 overexpression in the control CD4+ T cell population decreased miR-107 expression and increased STAT3 transcriptomic/proteomic presence, while the MEG8 silencing led to opposing outcomes (Figure 4E, 4F). RAOEC cells were utilized to elucidate MEG8/miR-107 and between STAT3/miR-107 (Figure 5A–5D). We found that miR-107 mimic transfection effectively upregulated miR-107 while significantly downregulating the STAT3 transcriptomic or proteomic content in the control CD4+ T-cell group. Thwarted miR-107 expression by miRNA inhibitors exerted an opposite effect on STAT3 expression (Figure 5E, 5F). These data indicated that MEG8 can upregulate STAT3 expression.

### The overexpression of MEG8 reversed the imbalance of the Th17/Treg ratio

**Figure 6:**
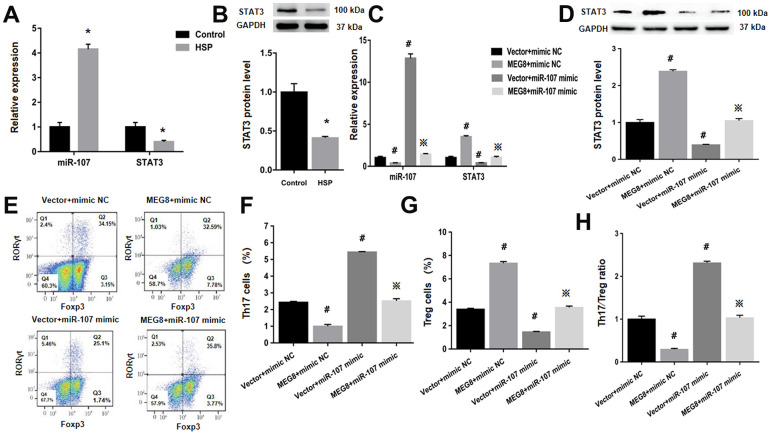
**MEG8 overexpression rescued the dysregulated Th17/Treg ratio by thwarting miR-107 within HSP CD4+ T-cell populations.** (**A**, **C**) miR-107 and STAT3 expression and (**B**, **D**) STAT3 protein level, (**E**–**G**) analysis of Th17 and Treg cellular populations, and (**H**) Th17/Treg cell ratio within HSP CD4+ T cellular populations simultaneously transfected with the mimic NC/miR-107 mimic and empty vector/MEG8 overexpression vector. *P <0.01 vs. Control group; ^#^*P <*0.01 vs. Vector + mimic NC group; ^※^*P <* 0.01 vs. MEG8 + mimic NC group.

**Figure 7:**
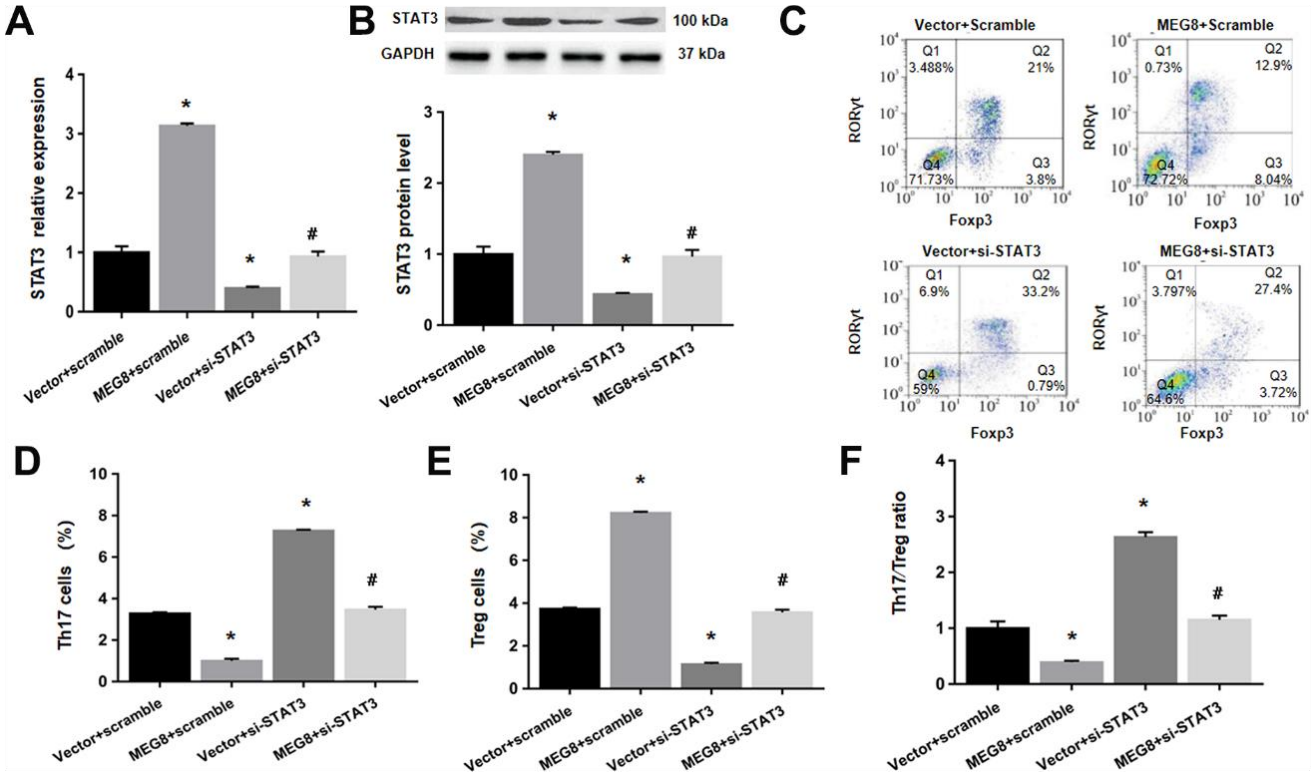
**MEG8 overexpression reversed the Th17/Treg ratio imbalance by upregulating STAT3 within HSP CD4+ T cellular populations.** (**A**) STAT3 mRNA level, (**B**) STAT3 protein level, (**C**–**E**) analysis of Th17 and Treg cells, and (**F**) the Th17/Treg cell ratio in HSP CD4+ T cells simultaneously transfected with the empty vector/MEG8 overexpression vector and scrambled siRNA/si-STAT3. *P <0.01 vs. Vector + scramble group; ^#^*P <*0.01 vs. MEG8 + scramble group.

Next, whether MEG8 can rescue Th17/Treg ratio imbalance through miR-107/STAT3 network in HSP CD4+ T cells population was determined. This study demonstrated that expression level of miR-107 was higher compared to the control cells (Figure 6A). On the other hand, levels of protein and STAT3 mRNA were lower in HSP CD4+ than controls (Figure 6A, 6B). Furthermore, MEG8 overexpression decreased miR-107 expression but increased levels of protein and STAT3 mRNA. But the phenomenon was not observed when miR-107 expression was activated through miR-107 mimic (Figure 6C, 6D). What matters is, miR-107 mock transfect suppressed HSP CD4 + T cells Treg differentiation, such as Th17 increase in cell number (Figure 6E, 6F), Treg cell ratio decreased (Figure 6E–6G), and Th17/Treg cells increased proved ratio compared to the simulated NC group (Figure 6H). In addition, the expression of miR-107 in HSP CD4+ T cells eliminated Treg differentiation caused by MEG8 overexpression (Figure 6E–6H). Similarly, STAT3 silencing attenuated the up-regulation of STAT3 levels (Figure 7A, 7B) and Treg differentiation (Figure 7C–7F) mediated by MEG8 overexpression in HSP CD4+ T cells. In summary, these findings indicated that MEG8 overexpression reversed Th17/Treg ratio disequilibrium through the miR-107/STAT3 network within HSP CD4+ T-cell population.

## DISCUSSION

LncRNA has a length of >200 nucleotides and lacks a protein-coding function. However, an in-depth investigation identified a regulatory role of lncRNA in biology and human pathogenesis [13]. LncRNAs functionally regulate multiple linkages in the immune system with a specific role within rheumatoid-immune condition pathogeneses [14]. Nonetheless, MEG8 involvement within HSP is yet unclear. Previously, we found that MEG8 was expressed at low levels in RMDM-enhanced M1 macrophages polarization within HSP rats [15]. Further experiments in this research demonstrated that MEG8 reverses Th17/Treg imbalance within HSP CD4+ T-cell population, thereby providing novel insights into the MEG8 mechanism of action in HSP.

Recent investigations revealed that Th17 cells promote inflammation responses together with having pivotal parts within autoimmune disease pathogenesis with anti-infectious processes [16]. However, Tregs inhibit inflammatory responses by limiting the excessive response of T cells, thus eventually maintaining immune tolerance [17]. The Th17/Treg imbalance is a key factor for multitudinous immune-related diseases pathogenic manifestations [18]. Interestingly, in rheumatoid arthritis (RA), Treg cells were decreased, whereas Th17 cells were increased [19]. Consistently, we observed increased Th17 cell number, decreased Treg cells, and an elevated Th17/Treg ratio within HSP CD4+ T cell populations.

Foxp3 is a specific transcription factor of Treg that inhibits the function of RORγt and enhances the nondifferentiation of Th17 cell populations. Consequently, this led to Th17-cell downregulation [20]. Furthermore, Treg cell populations limit Th17 cellular activities and even kill Th17 cells automatically by various molecular mechanisms [21]. Treg cell-produced IL-10 inhibits Th17 cell–mediated inflammation by enhancing the regulatory function of Foxp3^+^ Treg and TR-1 cells. IL-10 produced by Treg-cell inhibits Th17 cell-mediated inflammation by enhancing the regulatory function of Foxp3+ Treg and TR-1 cells. IL-10 can also inhibit Th17 cell-mediated inflammatory response at other levels. Tregs can kill Th17 cells by their cytolytic molecules [22]. Moreover, Th17 cells can synthesize and secrete IL-6, which mediates the differentiation and reduces the activity of Tregs, thus weakening the overall immunosuppressive function [23]. In the current study, MEG8 overexpression rescued Th17/Treg ratio homeostasis within HSP and rescuing Treg cells content and Foxp3 expression. These findings implied the immune regulatory role of MEG8, which needs to be substantiated further.

The advanced studies on lncRNAs have confirmed that lncRNAs exert a critical role in gene regulation, chromosome replication, and RNA splicing [24]. As a vital part of the immunoregulatory network, the gene expression of CD4+ T cells stimulated by antigens is regulated by lncRNAs [25]. Various studies on gene knockout and RNA interference have confirmed that lncRNAs have crucial roles in the differentiation, activation, and functional regulation of CD4+ T cell population [26]. Delving deeper into unraveling correlation between lncRNAs and the differentiation of CD4+ T-cell subpopulations in rheumatic immune diseases could underlie the pathogenesis and targeted therapy for these diseases [27]. Accumulating evidence has shown that lncRNA-p21 could partially isolate and inhibit the activity of NF-κB p65 to achieve an anti-inflammatory effect and alleviating RA. This phenomenon also indicated that lncRNAs regulate RA progression by affecting the differentiation of Th1/Th2 and Th17 cells with respect to CD4+ T-cell subsets [28]. In addition, the lncRNA V-maf has characteristics similar to that of Th1 cells. It inhibits the expression of the transcription factor MAF-4, thus promoting T-cell differentiation into Th1 cellular populations, and forming part of the pathogenesis of multiple sclerosis [29]. Xu et al. [30] demonstrated that the lncRNA miIR-155HG was highly expressed within serum of systemic lupus erythematosus (SLE) patients, could be used as a putative drug target in SLE. Moreover, mIR-155HG contains the precursor sequence of miR-155. MiR-155 overexpression upregulates IL-21, affecting the occurrence and development of SLE patients via Th17/Treg imbalance. Moreover, lncRNA AK124826 upregulation stimulates mitogen-activated protein kinase (MAPK) and STAT3 pathways and regulates Th17/Treg cellular differentiation. These phenomena eventually CD4^+^CD45RA^+^ T cell differentiation into Th17 cellular populations and inhibit Treg cell activity, thus underlying the pathogenesis of multiple dermatomyositis [31]. In the present study, MEG8 was a sponge that reduced the binding of miR-107, leading to upregulated STAT3 expression. Treg differentiation induced STAT3 is known primarily by binding to the Foxp3 gene promoter since it is regulating the activity of Treg cell signature and key transcription factors [32]. In summary, the current research results show that MEG8 attenuates miR-107-driven bespoke thwarting of STAT3/STAT3-based Treg differentiation through the miR-107 sponge action, which is a novel identified ceRNA regulating axis network in HSP rats.

MEG8 as a ceRNA may increase the expression of STAT3 by miR-107, thereby restoring the Th17/Treg imbalance rats. Thus, this study confirmed HSP has a putative therapeutic target.

## MATERIALS AND METHODS

### Animal model

In this study, Wistars rats aged 10–12 weeks procured from the Animal Research Center of Harbin Medical University (Harbin, China), were weighed (in the range of 250-300 g). The animals were intraperitoneally injected with 10 mg of alum and 1 mg of ovalbumin (Sigma-Aldrich, USA) in 1 mL of saline weekly (14 days in total) to establish the HSP model. Then the experimental cohort was intravenously treated with 15 mg/kg of ovalbumin and 1 mL of 0.3% ovalbumin through the external jugular vein, and 1 mL of normal saline was injected intracutaneously at 5 locations. The control rats were administered the same volume of normal saline. Subsequently, 40 mg/kg of pentobarbital sodium (Sigma-Aldrich, St. Louis, MO, USA) was given to the rats as anesthesia before the collection of blood samples from the posterior orbital venous plexus of the inner canthus [12]. According to the 3R criteria in animal experiments, all the animals were euthanized by intraperitoneally injecting them with 150 mg/kg of pentobarbital sodium.

### Isolation/culturing/T-cell transfection

The control and HSP CD4+ T cells were extracted from rat monocyte–derived macrophages (RMDMs) by using anti-CD4-conjugated magnetic beads and a MACS column (Miltenyi Biotec, Auburn, CA, USA) and grown within T-cell culturing medium (Gibco) at 37° C 5% carbon dioxide. The overexpression vector (pcDNA3.1-MEG8), empty pcDNA3.1 vector, MEG8 short-interfering RNA (siRNA; si-MEG8), signal transducer and activator of transcription-3 (STAT3) siRNA, scrambled siRNA, miR-107 mimic, miR-107 inhibitor, mimic negative control (NC), and inhibitor NC were all procured through Ribo Biotechnology (Guangzhou, China). The rat T-cell nucleofector kit and Amaxa nucleofector kit (Gibco, USA) were used to transfect both the HSP CD4+ T cells and control cells. The transfected cells were harvested after 48 h.

### Luciferase reporter assay

NR8383 and RAOEC cells (Sigma-Aldrich, St. Louis, MO, USA) were simultaneously transfected using the miR-107 mimic or mimic NC and pGL3-MEG8 wild-type (WT) or pGL3-MEG8-mutated (Mut) luciferase construct vectors (Miltenyi Biotec, Auburn, CA, USA), respectively. Luciferase activity was analyzed 24 h post-transfection by using the luciferase report analysis system (Promega, Madison, WI, USA).

### Estimation of Th17 and tregs

The cellular populations were consequently phosphate-buffered saline, (PBS)-washed, placed into incubation with FITC-labeled anti-CD4 (Solarbio Life Sciences, Beijing, China), and then fluorescently labeled monoclonal antibodies, (mAbs), PE-conjugated RORγt (BD Bioscience, La Jolla, CA, USA) and PE-conjugated Foxp3 (eBioscience, San Diego, CA, USA), in the dark (at 4° C) for 40 min. Then, the cellular populations were assessed on FACS Canto II flow cytometry (BD Bioscience, La Jolla, CA, USA), with data analyzed through FlowJo software.

### Real-time quantitative polymerase chain reaction

RNA was collected through RNeasy Plus Mini Kit (Invitrogen, Waltham, MA, USA), in line with kit-manufacturer protocol. cDNA was prepared through the PrimeScript RT Master Mix kit (TaKaRa Biotechnology, Tokyo, Japan). RT-qPCR runs were carried out in a 20-μL reaction consisting of cDNA (1 μL), 2× MasterMix (10 μL), 2 mmol/L of forward and reverse primers (1 μL each), and nuclease-free water (6 μL). PCR amplification reaction consisted of: denaturation at 95° C for 10 min, followed by 40 cycles of 95° C for 10 s, 60° C for 20 s, and 72° C for 15 s. All molecular genes of interest were analyzed (relative quantification) using the 2^−ΔΔCt^ analytical model, with *GAPDH* (TaKaRa Biotechnology) as the internal control. The primers are listed in Table 1.

**Table 1:** Primers used in the study.

**Genes**	**Primer sequences**	**Annealing temperature (° C)**	**PCR product (bp)**
GAPDH	sense: 5′-TCGCCAGCCGAGCCACAT-3′	60	149
	anti-sense: 5′-GGAACATGTAAACCATGTAGTTG-3′		
MEG8	sense:5′-CATCTAGACCCGTAACGCCC-3′	60	135
	anti-sense: 5′-CATTCCTCGGGTGTGGAGAC-3′		
STAT3	sense: 5′-ATGCGTACGTACGTATCGTGAT-3′	60	101
	anti-sense: 5′-ATCGATCGATCGTACGTAGTCG-3′		
miR-107	sense: 5′-CGAGAGGTAACATTCAACGCTGTC-3′	60	96
	anti-sense: 5′-GTGCAGGGTCCGTGGA-3′		
ROR-γt	sense: 5′-GCCAAGGCTCAGTCATGAGA-3′	60	116
	anti-sense: 5′-CCTCACAGGTGATAACCCCG-3′		
Foxp3	sense: 5′-GCCAAGGCTCAGTCATGAGA-3′	60	142
	anti-sense: 5′-CCTCACAGGTGATAACCCCG-3′		
U6	sense: 5′-CACTGGGCCATGCTAATCTTCTC-3′	60	89
	anti-sense: 5′-GTGCAGGGTCCGAGGT-3′		

### Immunoblotting

RMDM cell lysis was conducted using RIPA (Radio Immunoprecipitation Assay) buffer (Beyotime, Shanghai, China), with proteomic content evaluated through BCA^®^ (Beyotime). Equal proteomic loads were segregated through 1% SDS-PAGE (Beyotime) consequently being placed onto PDVF (polyacrylamide gel electrophoresis, polyvinylidene fluoride) membranes (Beyotime). Subsequently, 5% skimmed milk-blocking of membranes was performed, the buffer is 1*TBST, followed by primary antibodies y-set incubation (at 4° C overnight and incubated with corresponding horseradish peroxidase–conjugated secondary antibodies, the incubation conditions are 37° C, 2 hours. The immunoreactive bands were visualized using an ECL detection reagent (Seven Seas Biotechnology, China) and analyzed through Gel-Pro-Analyzer (Media Cybernetics, Los Angeles, CA, USA).

### Statistical analysis

SPSS v22.0 was employed throughout such analyses. Two-variable discrepancies were comparatively analyzed through unpaired Student’s t-test, while the multiple group discrepancies were evaluated through one-way analysis of variance (ANOVA). Datasets were represented as mean ± standard deviation (SD), and *P* < 0.05 indicated statistical significance for any discrepancies.

### Availability of data and materials

The datasets used and/or analyzed in this study are available from the corresponding author upon reasonable request.
